# Establishment of a Colony of *Phlebotomus argentipes* under Laboratory Conditions and Morphometric Variation between Wild-Caught and Laboratory-Reared Populations

**DOI:** 10.1155/2020/7317648

**Published:** 2020-03-30

**Authors:** Tharaka Wijerathna, Nayana Gunathilaka, Kithsiri Gunawardena

**Affiliations:** Department of Parasitology, Faculty of Medicine, University of Kelaniya, Kelaniya, Sri Lanka

## Abstract

The field-based studies on sand flies are not adequate to uncover information required for the control of the leishmaniasis through reduction of vector populations. Therefore, establishment and maintenance of laboratory colonies of sand flies is an essential step in leishmaniasis research. In the current study, a colony of *P. argentipes* was established from wild-caught sand flies following standard procedures from the published literature. Morphological measurements of laboratory-reared and wild-caught individual sand flies were compared to assess the difference between two groups. The colony was successfully established under confined laboratory conditions. The comparison of morphometric parameters revealed that the laboratory-reared sand flies are significantly larger than those caught from wild, suggesting a possibility of increased fitness of sand flies under favorable environmental conditions which may cause higher prevalence in the disease. The current study reports the first successful attempt in colonizing sand flies under laboratory conditions. However, the colony data suggest that the conditions extracted from the published literature need to be optimized to suit local settings in order to achieve maximum population sizes within the available amount of resources.

## 1. Introduction

Phlebotomine sand flies are medically important insects which are responsible for the transmission of leishmania parasites [1] and some other viral infections [2]. Leishmaniasis is a newly established disease in Sri Lanka with the record of more than 1000 patients annually. This could be considered as a significant health consideration throughout the island. Although a total of 20 species have been recorded in Sri Lanka, the only confirmed vector for disease transmission is *Phlebotomus argentipes*, which has been reported from almost all the endemic regions for the disease [3–9]. Many studies have reported that this species is the main vector for visceral leishmaniasis (VL) caused by *Leishmania donovani* in Indian subcontinent [10–12].

Laboratory-based studies on sand flies are vital to unveil the information required for disease control. Although field-based studies may reveal some valuable information on sand flies, some information such as vector parasite interactions, vectorial capacity, and host preference cannot be assessed through field-based observations. Therefore, rearing of disease vectors under confined laboratory conditions is essential in investigating the above aspects.

An ample amount of published information on techniques for rearing of sand flies at different settings and regions are available [13, 14]. These reports indicate conditions for the rearing of different species of sand flies. However, none of the published information reports the successful colonization of sand flies under laboratory setup in Sri Lanka.

The morphology of Phlebotomine sand flies including *P. argentipes* has been reported by several studies [15–17]. Although these reports provide valuable information to identify each stage of sand fly life cycle, identification of each instar of sand fly larvae still remains unclear. The variation of morphometric parameters between wild-caught and laboratory-reared individuals of the same species is documented previously for fruit flies [18, 19], which reports no significant difference between two populations. However, no attempt has been made previously to compare the morphometric variation between wild-caught and laboratory-reared individuals of sand flies.

In order to use for techniques, such as SIT, IIT, and paratransgenic approach, the laboratory-reared individuals should have proper physiological and biological features. The longevity, competitiveness, and reproductive capacity must be higher. Hence, the current study is focused on the initial establishment of sand fly rearing facility. Furthermore, the morphological and morphometric characterization of immature stages of sand flies along with morphometric evaluation of selected parameters between laboratory-reared and wild-caught individuals were also included.

## 2. Method

### 2.1. Field Collection of Sand Flies

Sand flies were collected from three sites (Galgamuwa, Maho, and Polpithigama) in Kurunegala District, using three standard entomological techniques, namely cattle-baited net trap (CBNT), light trap (LT), and outdoor hand catch (HC), using a battery operated aspirator. The collected sand flies were housed in insect cages until the transportation to the insectary facility at the Department of Parasitology, Faculty of Medicine, University of Kelaniya, Ragama, Sri Lanka.

### 2.2. Housing of Adults and Feeding Procedure

**Figure 1:**
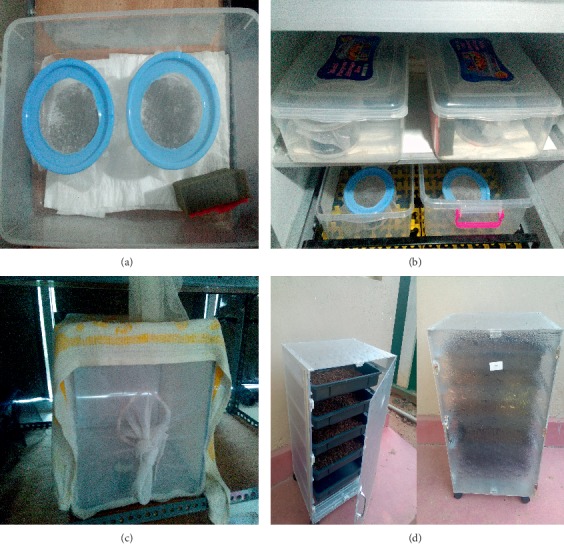
Equipment used for sand fly colony maintenance. (a) Larval rearing cups. (b) Egg laying cups and larval rearing cups placed in plastic boxes are maintained under standard conditions in an incubator. (c) Adult rearing cages made of white organdy clothes. (d) Larval food composter made for the fermentation of larval food.

A total of 200 sand flies (1 : 1 male to female ratio) were housed in insect rearing cages (15 × 15 × 15  cm), which were made of white nylon organdie supported by a steel frame of the same size (Figure 1). Field-caught unfed female individuals were taken for the present experiment. Adults were maintained under standard laboratory conditions (26 ± 1°C; 75–80% relative humidity (RH); a photoperiod of 12: 12  h (L : D)) with 10% sucrose solution provided *ad libitum*. Females were given a blood meal (*vide supra*) of cattle origin using an artificial metal plate membrane feeding method described by Gunathilaka et al. [20]. In this study, a square shaped metal plate with a rough surface (9  cm × 9  cm: width × length) was used. The rough surface of the metal plate was covered with a stretched Parafilm-M membrane keeping one side open. A volume of 3.0  ml of blood (cattle) was injected into the space between the plate and the membrane. The open side of the parafilm was carefully sealed. A small pillow (10  cm × 10  cm: width × length) filled with rice grains was heated to 40°C using a microwave oven and was placed on top of the metal plate, while keeping the blood-filled surface down in order to maintain the temperature throughout the time of blood feeding.

### 2.3. Oviposition of Sand Flies

Blood-fed female individuals were transferred into plaster of paris lined pots (400  ml) for larval rearing (Figure 1). The pots were moistened with water prior to the introduction of engorged females. The posts were kept at rectangular plastic container with tight fitting lid and humidity was maintained by spraying water daily and wet sponge placed inside the plastic container. The females were provided with 10% sugar and vitamin B solution *ad libitum* using saturated cotton wool pads during the oviposition period.

### 2.4. Species Identification of Sand Flies

Dead females were removed from oviposition cups using a fine paint brush or/and a fine forceps and placed in a 1.5  ml microcentrifuge tube. The specimens were placed 5  minutes each in 70% alcohol, 90% alcohol, absolute alcohol, and finally in xylene for dehydration. The dehydrated specimens were cleared by keeping in lactophenol solution overnight and placed on a slide with a few drops of the Hoyer medium and dissected it by removing the head, one wing, and the posterior segments of the abdomen (last 3–4). Flies were identified up to species level using available morphological identification keys to confirm that all the specimens are *P. argentipes* [21–23].

### 2.5. Egg Harvesting and Treatment

The egg pot was slanted to a cloth filter and eggs were removed from plastered substrate in the oviposition cup by spraying distilled water. The transferred eggs were subjected to egg treatment procedure. The filter with eggs was placed in 1% bleach for 2–3 minutes followed by 70% ethanol for 2–3 minutes in order to prevent gregarine parasitic infections to the eggs. The eggs were rinsed 3 times with distilled water and distributed among larval rearing pots. A little amount of warm water was added on to the eggs and kept until the eggs are hatched.

### 2.6. Larval Food Preparation

The equal amounts (1 gallon) of rabbit feces and rabbit food were put into a plastic basket and mixed thoroughly. A gallon of hot water was introduced into the basket while mixing. The mixture was kept for 15–20 minutes and distributed equally into 4 trays in the larval food composter. The bottom most tray was filled with hot water and trays containing the mixture were sprayed with warm water using a hand sprayer until the medium becomes shiny (no standing water). The lid of the food composter was closed and kept two vents opened in order to maintain aerobic condition. This setup was kept for one week, and the mixture was overturned at the end of that week and kept for another week followed by one week for drying.

### 2.7. Larval and Pupal Rearing

Following egg treatment, eggs were placed in larval rearing cups. When the larvae started to hatch, a thin layer of food was sprinkled over them. The larval rearing containers were mixed daily to provide a good aeration. The containers with pupae were left with minimum disturbance. The newly emerging adult sand flies were transferred to separate holding cages for 2–3 days prior blood feeding. The immature stages were maintained at the insectary at 26 ± 1°C, 95–100% RH, and 12 : 12 (L : D) photoperiod. The complete guide of sand fly rearing is included in [Supplementary-material supplementary-material-1].

### 2.8. Rearing Conditions and Safety Measures

All laboratory safety procedures as specified by the World Health Organization [22] were strictly followed to prevent occupational risks and escape to outside environment.

### 2.9. Duration of Larval Stages

Daily observations were made at each rearing container. The duration of all life stages starting from eggs, each instar of larvae, pupae, and adults were recorded, respectively. Morphological features that can be used to distinguish the larval stages of sandflies were noted referring to the exuviae of each larval stage.

### 2.10. Oviposition Rate, Hatching Rate, and Emergence Rate

Daily observations were made to determine oviposition rate, hatching rate, and the emergence rate. Oviposition rate was calculated as the number of eggs laid per female and hatching rate was considered as the number of hatched eggs as a percentage of the total number of eggs laid. Furthermore, emergence rate was defined as the number of emerged adults as a percentage of total number of eggs hatched.

### 2.11. Morphometric Measurements

**Figure 2:**
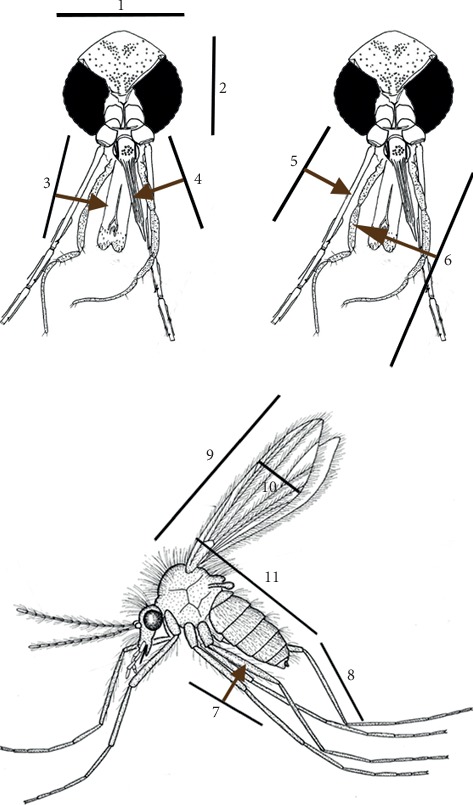
Morphometric parameters used to compare wild and laboratory-reared adult sand flies 1, head length; 2, head width; 3, labium; 4, labrum; 5, A3 length; 6, palp length; 7, hind femur length; 8, hind tibia length; 9, wing length; 10, wing width; 11, total body length.

All the measurements were taken under ×4, ×10, and ×40 magnification depending on the target structure. Morphometric parameters were selected based on the World Health Organization manual and Lawyer et al. [17, 22]. In order to compare the size variation of different life cycle stages, 25 randomly selected specimens of the life cycle stages (eggs, larval stages, pupae, and adults (male and female)) of the sand fly reared under laboratory conditions were measured using a precalibrated micrometer inserted in one of two 10 x ocular eyepiece lens of a light microscope (Olympus, CHT 4D0213). Total length was taken as the standard size measurement following Lawyer et al. [17]. For the comparison of size variation between wild-caught and laboratory-reared populations, morphometric parameters of the selected structures of adult sandflies namely head length, head width, labium, labrum, length of the 3^rd^ antennal segment (A3 length), palp length, hind femur length, hind tibia length, wing length, wing width, and total body length (Figure 2), which are the common characteristics for both genders were identified. Above parameters were measured using 50 randomly selected adults with 1 : 1 sex ratio (25 from each sex) from both wild-caught and laboratory-reared populations.

### 2.12. Data Analysis

Univariate statistical analysis of morphometric parameters between wild-caught and laboratory-reared populations was carried out in IBM SPSS 25 statistical software package. The significance of the mean difference of measured characters between two populations was evaluated using the independent samples *t* test at 95% confidence intervals. Multivariate statistical analysis was carried out using principal component analysis (PCA). The PCA scores were computed, and the PCA scatter plot was generated using vector products of original data. PCA was carried out in *R* statistics 3.6.2 program. The scores of PC1 and PC2 of each population were compared using Student's *t*-test.

## 3. Results

### 3.1. Duration of Life Stages and Development Characteristics

A colony of *Phlebotomus argentipes* was successfully established under confined conditions. The adult female sand flies started laying eggs 6 days after blood meal and lasted for 8 days. Egg hatching was started 3–5 days after oviposition. Life span of immature stages of the sand flies in the colony was 31–33 days. Larval stage lasted for 18–24 days, and the duration of pupal stage was 8–9 days. The average life span of emerged adults was observed as 7–8 days for males and 9–11 days for females. Total development period from egg laying to adult stage ranged from 36 to 50 days (Table 1). The blood feeding rate of female individuals was 100%. However, oviposition rate of females were 10.03/female out of 100 females used for the experiment followed by a hatching rate of 76.57% (*n* = 768). Pupation success in the present trial was 46.10% (*n* = 354), and all of them were successfully reached to the adult stage.

### 3.2. Morphometry and Morphology in Different Life Cycle Stages of Laboratory-Reared *P. argentipes*

**Figure 3:**
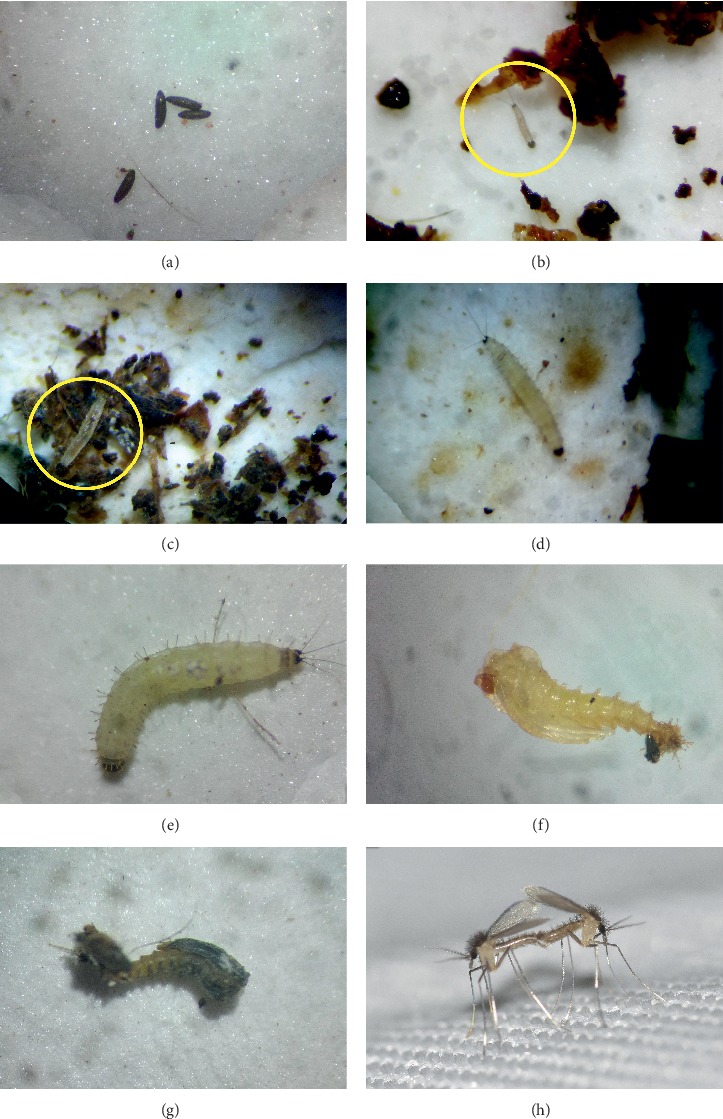
Life stages of *Phlebotomus argentipes*: (a) eggs, (b) L1 larva, (c) L2 larva, (d) L3 larva, (e) L4 larva, (f) early stage of pupa, (g) pupa few hours before emergence, and (h) adult sand flies in a cage.

The *P. argentipes* eggs were elliptical in shape and brownish black in color (Figure 3(a)). Eggs were approximately 0.33–0.36 mm in length (Table 1). The larvae were caterpillar like and whitish in color. Body contains lateral setae during all four instar stages. The head capsule was black in all instars (Figure 3). The 1^st^ instar larva could be identified separately by very small body size ranged from 0.64 to 0.91  mm and having only one pair of caudal filaments (Figure 3(b)), since all other instars had two pairs of caudal filaments.

The second instar larvae (Figure 3(c)) were obviously larger than first instar which ranged from 1.12 to 1.55  mm in length (Table 1) and the third instar larvae (Figure 3(d)) ranged from 2.11 to 2.44  mm in length (Table 1). Fourth instar (Figure 3(e)) was even larger and ranged from 3.06 to 4.08  mm in length (Table 1). The presence of a characteristic dark colored, sclerotized area on the dorsal side of the penultimate segment of the fourth instar larvae is useful to separate it from other stages. However, only the third and fourth instar stages are clearly visible to the naked eye. Pupa had a shiny white color on the first day after pupation, but later turned to a yellowish color (Figure 3(f)). Approximately 1–2 days before emergence, pupae were turned to a dark color. Body parts such as wings, head, and abdomen of adults were clearly visible through the pupal case (Figure 3(g)).

### 3.3. Morphometric Variation between Wild-Caught and Laboratory-Reared *P. argentipes*

Both univariate ([Supplementary-material supplementary-material-1]) and multivariate analyses suggested a significant difference in morphometrics between wild-caught and laboratory-reared populations of *P. argentipes*. The principal component analysis of 11 measured morphometric characters indicated that the first principal component (PC1) and second principal component (PC2) accounted for 74.66% and 6.69% of overall variance in two populations of *P. argentipes*. These factors accounted for 81.35% of overall variance when combined (Table 2).

The scores of PC1 of the laboratory-reared population and wild-caught population is significantly different (*t* = 7.84, df = 94, *P* < 0.001). Although the variance explained by PC2 is considerably lower, the difference between two populations was significant (*t* = 7.54, df = 97, *P* < 0.001). The correlation between original variables and the component scores were positive indicating that laboratory-reared sand flies are larger than the wild-caught sand flies.

**Figure 4:**
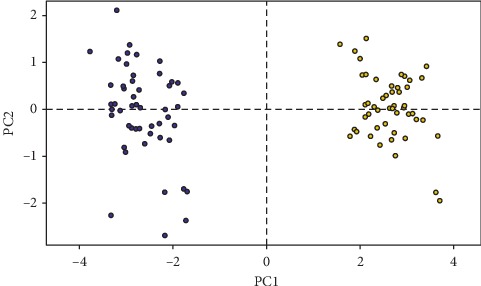
Principal component analysis (PCA) of wild-caught (blue color) and laboratory-reared (yellow color) *Phlebotomus argentipes*.

The first axis (PC1) was positively correlated to all the morphological factors except the length of the labrum, which showed a negative correlation. The second axis (PC2) was highly positively correlated to the length of the labrum (Table 3 and Figure 4).

The PCA scatter plot confirms the difference of two populations (wild-caught and laboratory-reared) based on morphometric parameters (Figure 4).

## 4. Discussion

The current study reports the first successful colonization of sand fly species under laboratory conditions in Sri Lanka. The gradual adaptation of the wild-caught sand flies to laboratory conditions was achieved by effective blood feeding using an artificial blood feeding technique and meticulous maintenance practices. The analysis of morphometric parameters via principal component analysis and subsequent *t*-test indicated that the laboratory-reared sand flies are much larger than the wild-caught sand flies.

In the establishment of the colony, wild-caught females were fed with cattle blood using an artificial membrane feeding method. In general, the duration of egg production was affected mainly by the time taken for blood digestion, which varies depending on the species [24]. Furthermore, some external factors such as temperature, humidity [25], and blood meal source are also important in determination of the egg production time [26, 27]. The oviposition commenced 6 days after blood meal in the current study, which agreed with previous reports [17, 26]. Some species such as *P. duboscqi* (8–10 days), *P. tobbi* (7–11 days), and *P. halpensis* reported to have much longer gonotrophic cycles than *P. argentipes,* while other species such as *Lutzomyia longipalpis* and *P. papatasi* have gonotrophic cycles similar to the duration of *P. argentipes* [26]. However, species specific factors may be the main reason for fast production of eggs and early stimulation for oviposition.

The ability of a species to oviposit within a short period after a blood meal is not the only factor for successful colonization of sand flies. The oviposition rate (the number of eggs laid per female) should also be higher for a better colonization [26]. In general, the number of eggs laid by a female ranges between 30 and 70, depending on the species and the nutritional condition during larval and adult rearing [26]. This lower oviposition rate observed during the current study may be because of the poor living conditions including nutrient deprived soil in natural habitats. Source of blood meal and the efficiency of blood feeding method may also influence oviposition rate. When the blood feeding success of *P. argentipes* is concerned, it has proven to be a very poor feeder when anesthetized mammals were used [28]. In this study, 100% blood feeding rate was achieved by using the metal plate membrane feeding method described by Gunathilaka et al. [20]. Therefore, it could be determined that the method of blood feeding is not a reason for the lower oviposition rate. Hence, higher oviposition rates may be achieved by optimizing the blood source and feeding method, which should be performed once the colony is established.

In the mass rearing, larval diet quality and rearing conditions have a direct and often irreversible effect on adult traits of insects [29–32], especially on the size of the laboratory-reared adults and other physiological functions associated with their nutrition. Therefore, the higher body size observed among the second generation of laboratory-reared colony is probably due to enhanced conditions and nutritional diet provided during larval rearing. In addition, there is no disturb from predators, and other pathogenic agents in the rearing facility may also enhance growth and survival, which may be influenced as inhibiting factors for their growth in the natural environment.

In addition to other vector-borne infections, sand fly-transmitted diseases are becoming major public health issues in tropical countries with climatic changes occurring in nature. This work was carried out on establishment of laboratory colony of wild-caught *P. argentipes*, and its characterization is important for understanding the changes enforced by altered environment on the disease transmission vectors. This work may represent rare achievement in the context of research on vector-borne infections in Sri Lanka. However, to carry out research on biology of vector vector-borne disease, establishment of laboratory colonies are the prerequisite, and such colonies are established in most of the research organizations conducting research on vector–pathogen relationship. Therefore, the first ever approach to colonize sand flies in Sri Lanka will be a beneficial catalyst to enhance research activities towards evaluating modern vector control approaches, vector parasites interactions which have not investigated sufficiently in Sri Lanka.

One of the major issues in establishment of sand fly colonies is the high mortality during the larval stages [13]. The present study also suffered with the same issue, in which only 46.10% of the hatched larvae reached the adulthood under laboratory conditions initially. This may be due to the fact that the fungal growth in the medium, improper diets, or moisture and temperature conditions inhibits the larval development [13]. In order to find a remedy, the eggs harvested from the oviposition pots were subjected to egg treatment as a precaution for preventing fungal and gregarine infections. In addition, larval rearing containers were gently brewed everyday using a fine paint brush in order to prevent the growth of fungal threads which may trap sand fly larvae especially during the first instar stage. However, nonautoclaved larval food was used for larval rearing following Goulart et al. [33], since their findings suggest that species of genus *Phlebotomus* prefer nonautoclaved food. The diet and the rearing conditions were determined based on the previously published information. This initial colonization data suggest that it is essential to optimize the larval procedures and rearing conditions with minimal larval mortality.

At the end of fourth instar stage, larvae stop feeding and start development into pupa by forming prepupa. Although, some temperate species tend to diapause naturally at the fourth instar stage, these tropical sand flies do not exhibit such behavior. However, the presence of pathogens and other unfavorable conditions may result in the diapause of fourth instar larvae. Although the duration of the fourth instar larvae is slightly higher than other stages, a diapause of the fourth instar larvae was not observed under current conditions.

The emergence of sand flies occurs in two patterns: first is synchronous emergence where all the adults emerge within two or three days, and the second one is semi-synchronous emergence where the emergence period lasts for several weeks to several months [17]. The current study showed that the species *P. argentipes* shows synchronous emergence pattern under laboratory conditions.

Although several studies have attempted in describing the larval morphology of sand fly larval, most studies had not provided exact morphological features and measurements at each larval instar. The current study provides descriptive information which is valuable for grading of larvae into instar. The sizes of selected morphometry among different instars were within the ranges indicated in previous publications [17]. Some attempts have been made by researchers in order to evaluate the morphometric variation between isolated populations either through geographic, temporal, or man-made barriers [18, 34–37]. The present study revealed that the laboratory-reared *P. argentipes* were significantly larger at 95% significant intervals than the wild-caught individuals.

The first principal component (PC1), which explains 74.66% of the overall variance, is largely affected by A3 length, palp length, length of hind leg segments, head parameters, and labium. The coefficient values were positive for all parameters except for the length of the labrum, indicating a higher value for the length of labrum in wild-caught populations. The opposite is true for other morphometric parameters where the size is higher in laboratory-reared populations. The variance explained by PC2 is considerably lower when compared to PC1, but the difference is considerably high as indicated by the *t*-test. The PC2 is affected only by the length of labrum indicating a positive coefficient value. This further confirms the significantly larger labrum size of wild-caught populations. However, in summary, the results suggest that the laboratory-reared sand flies are much larger than the wild-caught sand flies.

A comparison of laboratory-reared and wild-caught sand fly in terms of morphometric characters was not reported previously. However, some studies have attempted in evaluating the morphological distinctiveness of four populations of *P. papatasi* living at different altitudes [35]. This study has reported a significant variation between attitudinally separated populations, which has been advocated by another study conducted using *P. ariasi* [38]. The nutritional content in the soil for developing larvae which may differ at different altitudes could have also play a key role in this observed morphometric distinction [17]. The same could be the reason for the significant difference observed in the current study. The morphometric difference provides valuable input to plan future studies under several aspects. For instance, host skin penetration ability and depth of penetration of sand flies depend on the length of mouth parts [39]. This might be one of the reasons that the *L. donovani* causes mainly CL in Sri Lanka and visceral presentation in other South Asian countries since local sand fly vectors may be incapable to reach higher depth in the skin and thereby localized on skin [39]. Although there is a genetic variation among two strains of parasites identified from Sri Lanka and other South East Asian region, it is still unclear regarding the exact mechanisms and reasons for this difference in clinical presentation. Furthermore, the disease, which was previously prevalent in dry zone areas of Sri Lanka, is now spreading to wet zone areas, where much better environmental conditions are available [40]. Therefore, if better conditions in the environment affect the nature of the infection, disease may be more serious in wet zone areas by enhancing the parasitic inoculation ability due to variation in morphology. Hence, the elaboration of vector-related studies to identify any effects of sand fly morphometrics on enhancing the vectorial capacity, entomological inoculation rate, and disease transmission is highly recommended.

In the laboratory, it is important to follow extreme precautions to prevent the escape of any sand fly especially gravid females. When sand flies escape from cages, the immediate actions were taken to catch them using a hand held aspirator or kill them using a fly swatter.

A light trap was placed inside the lab to prevent sand flies from escaping to the outside of the laboratory. Corners and vertical surfaces of the lab were inspected daily for any escaped sand fly without noticing.

Overall, establishment of a sand fly insect colony from wild-caught individuals is difficult, time-consuming, uncertain, and challenging task since the developmental parameters of these species are still unknown. Thus requires extreme care and proper holding containers to ensure the survival of sand flies until the production of eggs. Another major problem is that the genetic bottleneck occurs in the laboratory population which causes the colony to collapse after few generations [17]. This makes it very difficult to maintain a closed colony without further input from wild. Also the infections including fungi, bacteria, gregarines, and some mites could become a major problem and may lead to the collapsing of the colony [26, 41]. Therefore, the established colony in the present study is maintained under maximum care with proper quality control procedures. Furthermore, studies will be carried out to optimize the rearing conditions related to larval food, oviposition substrates, concentration of feeding sugars for adults, optimum sex ratios in mating cages, and carrying capacities in holding containers in order to achieve the maximum productivity with the currently available facilities.

## 5. Conclusions

The colonization of *P. argentipes* was feasible under laboratory conditions in Sri Lanka with proper maintenance practices and extreme care. Furthermore, the laboratory-reared individuals of *P. argentipes* were larger than those caught from wild indicating the potential of increasing the fitness of the vectors under favorable environment conditions, which intern may increase the transmission of the disease.

## Figures and Tables

**Table 1:** Mean (±standard variation) values for longevity and size at each stage in F1 generation of laboratory-reared population *Phlebotomus argentipes* under confined conditions (26 ± 1°C, 75–80% relative humidity (RH), and a photoperiod of 12: 12 h (L : D)).

Stage	Duration (days)		Duration of development stages (Days)	Size (mm)		Range in size (mm)
	Mean	SD ±		Mean (*n* = 25)	SD ±	
Eggs	3.8	0.789	3–5	0.33	0.011	0.33–0.36
1^st^ instar larva	5.8	0.789	5–7	0.76	0.097	0.64–0.91
2^nd^ instar larva	4.2	0.422	4–5	1.29	0.120	1.13–1.55
3^rd^ instar larva	3.4	0.516	3–4	2.22	0.084	2.11–2.44
4^th^ instar larva	6.8	0.789	6–9	3.34	0.340	2.93–4.08
Pupa	8.4	0.516	8–9	2.41	0.074	2.26–2.54
Adults						
Male	7.4	0.516	7–8	2.89	0.183	2.43–2.98
Female	9.8	0.789	9–11	2.44	0.166	2.21–2.70

**Table 2:** The percent variance and cumulative variance of the axes from the principal component analysis of 11 morphometric measurements of wild-caught and laboratory-reared *Phlebotomus argentipes*.

Axis (principal component)	Percentage of variance (%)	Cumulative (%)
1	74.66	74.66
2	6.69	81.35
3	5.74	87.09
4	3.97	91.06
5	3.37	94.44
6	2.73	97.17
7	1.29	98.46
8	0.86	99.32
9	0.31	99.63
10	0.25	99.88
11	0.12	100.00

**Table 3:** Characterization of wild-caught and laboratory-reared populations using the loadings of principal component analyses on 11 morphometric variables.

Morphometric variable	Principal components	
	PC1	PC2
Head length	0.27439	0.11533
Head width	0.23800	−0.03821
Labium	0.28897	0.13788
Labrum	−0.19840	0.93904
A3 length	0.32505	0.13314
Palp length	0.33891	0.10048
Hind femur length	0.34129	0.10984
Hind tibia length	0.33952	0.09148
Wing length	0.27080	−0.17736
Wing width	0.32127	0.00877
Total body length	0.34114	0.068136

## Data Availability

All data are available with the authors and will be provided upon reasonable request.

## Abbreviations

WHO: World Health Organization
CBNT: Cattle-baited net trap
LT: Light trap
HC: Hand catch
PCA: Principal component analysis.

## Supplementary Materials

Supplementary Materials Supplementary 1: a brief guide to rearing of Phlebotomine sand flies. Supplementary 2: univariate analysis of morphometric variables between wild-caught and laboratory-reared *Phlebotomus argentipes* populations.
