# Supplementary material for: Establishment of a Colony of Phlebotomus argentipes under Laboratory Conditions and Morphometric Variation between Wild-Caught and Laboratory-Reared Populations
Source: J Trop Med. 2020 Mar 30;2020:7317648. doi: 10.1155/2020/7317648 (PMC7149484; doi:10.1155/2020/7317648)
Supplement: Supplementary Materials — Supplementary 1: a brief guide to rearing of Phlebotomine sand flies. Supplementary 2: univariate analysis of morphometric variables between wild-caught and laboratory-reared Phlebotomus argentipes populations. [file 7317648.f1.doc]

**Supplementary 1: A brief guide to rearing of Phlebotomine sand flies**

**Housing sand fly adults**

Transfer non-gravid and un-engorged sand flies into a suspended nylon cloth cages and provide a sugar feeding. Facilitate females to obtain a blood meal using an artificial blood feeding method as described below. Colony should be maintained under controlled conditions as specified below.

- Room with controlled temperature or in an incubator; 24-28° C
- Photo period; 14:10 (light:dark)
- High humidity (70-95%) should be ensured by wrapping cages in plastic bags with wet tissue or cotton wool inside
- Surrounding humidity in the insectary or incubator: 60-70%

**Initiation of a colony from field caught engorged females**

Separate blood fed Sand flies visible to the naked eye which capture through field surveys and artificially fed individuals using a mouth aspirator with a HEPA filter. Transfer engorged females into plaster-lined plastic pots used for larval rearing.

Keep the pots in plastic rectangular boxes with tight-fitting lids. Ensure the high humidity by a layer of moistened filter paper or tissue in the bottom of the box. Use a cotton wool pad saturated in 10-50% sucrose solution to maintain a continuous supply of sugar for sand flies. For most species, the optimum temperature is between 24-28°C. Pots in boxes are also useful for the transporting of flies, but cooler conditions (15-20°C) during transportation is recommended.

**Blood feeding of adult female sandflies by metal plate artificial membrane method**

- Prepare a square shaped metal plate with a rough surface (9 cm x 9 cm: width x length) and hot pad (10 cm x 10 cm: width x length) filled with heat resistant gels.
- Cover the rough surface of the developed metal plate with a stretched Parafilm membrane leaving one side open.
- Add 3.0 ml of blood into the space between the plate and the membrane, and seal the open side carefully.
- Place the blood filled metal plated on top of the insect adult rearing cage.
- Heat the heat pad up to 40 ^0^C using a microwave oven and placed on top of the metal plate, while keeping blood filled surface down in order to maintain the temperature throughout the time of blood feeding.
- After feeding, leave the females undisturbed for 12-24 h, mainly for complete formation of the peritrophic matrix, which makes their gut less fragile.

**Notes:** The presence of sand fly males may improve the engorgement rate.

Cover the cage with a dark tissue since females prefer feeding in the dark

- After laying the eggs, females die or could be killed for species identification. Transfer the pots with eggs for egg harvesting.

**Preparation of egg laying cups and larval rearing pots**

**Material:** Plastic cups with lids (300 ml), Plaster of Paris, 1 L plastic beaker, water, spatula

- Drill the bottom and the lid of the plastic cup (Do not remover the whole base and top)
- Fix a net on the on the lid of the plastic cup covering the removed part.
- Add equal volumes of plaster of Paris and water in to the 1 L beaker and mix thoroughly for 15 minutes (Add more water if necessary).
- Keep the plastic pot on a flat surface and fill with plaster of Paris mixture up to about 1-inch thickness.
- Keep the plastic pots for 24-48 hours in order to solidify the medium followed by soaking pots overnight dipping in a tray filled with water (facilitate to wet from bottom to top).

**Introducing blood fed female sand flies into egg laying cups**

- Obtain the wet egg laying cups as described above.
- Cover the top using a net material and tie using rubber bands
- Keep a small hole on the top to facilitate the transferring of sand flies
- Transfer 100 engorged females sand flies in the adult rearing cages into the egg laying cup using a battery operated aspirator.
- Cover the hole in the net using a cotton plug
- Barricade the cups in sterile plastic storage bins and keep the closed storage bins at the incubator

**Harvesting and treatment of eggs**

**Material:** 70% Ethanol, 1% Bleach, Distilled water, Trigger sprayer bottle, paint brush, sieve (0.22 μm disposable vacuum filter).

- Remove the carcasses in the oviposition cups by aspiration
- Slant the egg pot to the filter and spry the pot using water
- Put warm water (50^o^C) into the egg pot and introduce to the filter
- Transfer all eggs in the pot and follow egg treatment procedure

**Egg treatment**

Step 1- 1% bleach for 2-3 minutes

Step 2- 70% Ethanol for 2-3 minutes

Step 3- Rinse 3 times with distill water

Step 4- Distribute eggs among larval rearing pots

- Add a little amount of hot water and put in a plastic tray lined with a paper towel.
- Keep overnight and use for larval rearing.

**Preparation of sand fly larval food.**

**Material:** Rabbit feces (1 gallon), Rabbit food (1 gallon), Hot water (1 gallon), plastic basket, plastic trays, larval food composter

This is the most important step in the sand fly colonization.

- Put the equal amounts (1 gallon) of Rabbit feces and Rabbit food in to the plastic basket.
- Mix thoroughly and introduce 1 gallon of hot water in to the basket while mixing
- Keep the mixture for 15 – 20 minutes.
- Distribute the mixture equally in to 4 trays (Number of trays may vary based on the capacity of the food composter) and put in to the la1rval food composter
- Fill the bottom most tray with hot water.
- Wet the trays containing the mixture using a hand sprayer until the medium become shiny (No standing water).
- Close the lid of the larval food composter and keep the two vents open in order to maintain the aerobic condition during the digestion.
- Keep the setup for two weeks followed by 1 week for dry.
- Grind the prepare using an electric grinder and store in a plastic basket until the use.

***Note:***

****Flip the food using a spatula and break it up into small pieces after 1^st^ week.***

****Wet the trays again one-week after***

****Alternate the trays after the first week.***

****Introduce hot water into the bottom tray every week***

**Larval rearing and feeding**

- When the larvae start emerging from eggs, sprinkle a little amount of the larval food. Add more food as required, depending on the daily observations of the pots (If the larval excretory matter is more than 50%, add food and mix well)
- Wet the cups from bottom to top while dipping in a tray filled with water (Keep for 15 minutes)
- Fix the lid of the egg laying cup and barricade in a sterile storage bin.
- Keep the closed storage bin in the incubator (26 ^0^C, RH 75%- 80%).
- Mix the larval rearing cups daily in order to aerate the pots and break fungal threads.
- Once the larvae develop in to pupa and subsequently emerge as adults. Transfer the adults in to rearing cages.

**Supplementary 2: Univariate analysis of morphometric variables between wild caught and laboratory reared *Phlebotomus argentipes* populations**

Adult stage of *P. argentipes* of the laboratory reared colony was significantly larger than the size of wild population according to the independent t test (95 % significant intervals). The total body length, labium length, length of the antennal segment 3, length of palps, length of hind femur was significantly higher in laboratory reared population (*P*<0.001). The length of the hind tibia and width of the wing was significantly higher in laboratory population with (*P*<0.05). However, labrum length and wings length did not indicate a significant difference between two populations at 95 % significant intervals (Table 2).

**Table 2**. Morphometric variation between wild caught and laboratory reared populations.

| **Morphometric variable (µm)** | **Wild caught population (n=50)** | | **Laboratory reared population (n=50)** | | **t-test** |
| --- | --- | --- | --- | --- | --- |
|  | **Mean** | **SD ±** | **Mean** | **SD ±** |  |
| Head length | 350.0 | 17.5 | 391.0 | 47.1 | -3.65* |
| Head width | 419.0 | 53.6 | 446.0 | 15.4 | -2.17* |
| Labium | 260.0 | 24.1 | 285.0 | 11.0 | -4.23** |
| Labrum | 280.0 | 33.4 | 271.5 | 14.1 | 1.05 |
| A3 length | 215.5 | 15.7 | 244.60 | 7.87 | -7.42** |
| Palp length | 414.5 | 30.2 | 489.4 | 46.8 | -6.01** |
| Hind femur length | 727.5 | 15.5 | 863.5 | 43.0 | -13.32** |
| Hind tibia length | 1199.5 | 36.6 | 1375 | 238 | -3.25* |
| Wing length | 1804.5 | 62.1 | 1859 | 127 | -1.71 |
| Wing width | 540.0 | 18.1 | 593.0 | 57.1 | -3.96* |
| Total body length | 2058 | 137 | 2564 | 213 | -12.61** |

* p<0.05, ** p < 0.001
